# Sonographic reference values of median nerve cross-sectional area: a protocol for a systematic review and meta-analysis

**DOI:** 10.1186/s13643-018-0929-9

**Published:** 2019-01-03

**Authors:** Sandy C. Takata, Lynn Kysh, Wendy J. Mack, Shawn C. Roll

**Affiliations:** 1 Chan Division of Occupational Science and Occupational Therapy, University of Southern California, 1640 Marengo St, Suite 505, Los Angeles, CA 90089-9256 USA; 2 Norris Medical Library, University of Southern California, Los Angeles, CA USA; 3 Children’s Hospital Los Angeles, Los Angeles, CA USA; 4 Department of Preventive Medicine, University of Southern California, Los Angeles, CA USA

**Keywords:** Meta-analysis, Systematic review, Musculoskeletal sonography, Median nerve, Reference values

## Abstract

**Background:**

Median nerve cross-sectional area (CSA) is the primary sonographic parameter for assessing and diagnosing median nerve pathology, such as carpal tunnel syndrome. However, variability in the sensitivity of diagnostic thresholds exists, which may be due to a lack of standardized normative reference values. Current estimates of normal median nerve CSA stem largely from small studies using a local pool of healthy controls. A systematic review and meta-analysis will be conducted to identify all available data for median nerve CSA in healthy, asymptomatic individuals to create a comprehensive set of normative reference values.

**Methods:**

Articles that include sonographic measures of median nerve CSA will be identified through a rigorous search of published evidence, a hand search through tables of contents of key journals, and the gray literature, including ClinicalTrials.gov and conference abstracts. Each abstract and full text will be reviewed by multiple raters to identify studies from 2000 to present that include original data. Any study that provides median nerve CSA values from healthy individuals will be included (e.g., reference value study, control participants in a diagnostic study). Studies will be assessed for quality using a modified version of the National Institute of Health Study Quality Assessment Tool for Observational Cohort and Cross-Sectional Studies, with primary focus on the use of a detailed and acceptable image acquisition and analysis protocol. Using data from included studies, reference values will be calculated for median nerve CSA by anatomical regions, including the distal forearm, wrist, and carpal tunnel at the level of the pisiform. Reference values will be stratified by gender, ethnicity, and age based upon the specificity of the data provided by the included articles.

**Discussion:**

A comprehensive set of normative reference values of median nerve CSA will reduce variability across studies, allowing future research to more accurately evaluate and establish diagnostic thresholds. Additionally, normative values can serve as a reference for evaluating treatment outcomes and provide a means to investigate and understand minor nuances in CSA changes that may be indicative of preclinical stages of median nerve pathology.

**Systematic review registration:**

PROSPERO 2016 CRD42016037286

**Electronic supplementary material:**

The online version of this article (10.1186/s13643-018-0929-9) contains supplementary material, which is available to authorized users.

## Background

Carpal tunnel syndrome, a compression neuropathy of the median nerve, impacts 10 million people annually, costs employers up to $113,695 per incident, and is the most expensive upper extremity musculoskeletal disorder with an estimated cost of medical care in the USA exceeding $2 billion annually [1]. Musculoskeletal sonographic imaging is becoming increasingly used in the evaluation of the median nerve for diagnosis of carpal tunnel syndrome. Across the various options, measurement of median nerve cross-sectional area (CSA) has been shown to be the most useful sonographic parameter for tracking the development of median nerve pathology and conducting diagnostic evaluations; nerve CSA has also been shown to be positively correlated with severity of carpal tunnel syndrome [2–4].

Despite the promising and supportive data for the use of sonography in the evaluation of median nerve pathology, three significant concerns remain pervasive across research and clinical literature. First, considerable variability in diagnostic accuracy of sonographic imaging for carpal tunnel syndrome exists, with specificity and sensitivity ranging broadly from 78 to 87% [5–9]. This variability has hampered widespread implementation of sonography, especially as a replacement for electrodiagnostic testing for diagnosis of carpal tunnel syndrome [10, 11]. Second, measurement of CSA of > 10 mm^2^ has been primarily adopted as a minimum diagnostic threshold for carpal tunnel syndrome. However, evidence exists that this threshold may be either too high or too low to be clinically accurate, leading some studies to suggest sonography is best utilized for diagnosing severe cases with a much higher threshold (e.g., 14 mm^2^, 18 mm^2^) [3, 12]. Moreover, there remains debate as to whether a single threshold value versus a combination of factors is most appropriate [13], and questions arise as to which anatomic location should be used for the diagnostic CSA measurement [14]. Finally, there is limited guidance or understanding for how to best interpret findings in individuals who are in an early, preclinical state with symptoms, but normal test findings [15–18].

Much of the variability in the literature and the resulting inconsistencies in evidence stem from a lack of standardized reference values for the healthy median nerve. The vast majority of studies evaluating diagnostic accuracy and testing other parameters for the use of sonography for median nerve pathology recruit a unique healthy or asymptomatic control sample for use in analysis. Although these samples may be representative of the comparative participant sample within each individual study, the use of small selective samples likely contributes to the significant variability in outcomes across studies. A handful of studies have been conducted in an attempt to provide references values for median nerve CSA in certain sample populations [19–23]; however, these studies have yet to be widely used. Furthermore, these studies are often limited to a specialized population and may not be useful for all future research. The development of a comprehensive set of reference values that crosses various demographic populations would provide a foundation upon which future studies could be more systematically evaluated to advance knowledge and practice. To advance the precision of screening, prevention, and diagnosis for the evaluation of median nerve pathology, it is essential to have a robust set of common reference values for the CSA of the median nerve that can be used in research and clinical practice.

## Objective

A systematic review and meta-analysis will be conducted to identify a comprehensive set of reference values for the CSA of the median nerve in the distal upper extremity within pooled data, stratified across anatomical locations, age, sex, and ethnicity.

## Methods

This review protocol adheres to the requirements of Preferred Reporting Items for Systematic Reviews and Meta-Analyses Protocols (PRISMA-P) [24], and the final report will be developed to meet the requirements of the PRISMA guidelines [25]. The study was initially registered in PROSPERO, the International Prospective Register of Systematic Reviews, on April 3, 2016, (ref id: CRD42016037286) and was last updated November 28, 2017. The project is ongoing with the initial search completed and full-text screening in-progress. Future amendments and updates to the protocol will be documented through PROSPERO. A detailed description of the steps involved in this systematic review and meta-analysis protocol follows.

### Eligibility criteria

#### Study design

The data included in this review will be extracted from all published and non-published literature that report original, empirical data. Appropriate study designs include comparative/diagnostic trials or observational studies. Articles that are reviews of the literature and expert opinion or have other designs that do not report on primary data will be excluded.

#### Population of interest

This outcome of this review is meant to establish normative reference values; therefore, only studies reporting data from healthy, asymptomatic individuals will be included. Data from individuals with symptoms of median nerve pathology in the distal upper extremity, a diagnosis of carpal tunnel syndrome, or any other diagnosis associated with median nerve pathology will be excluded. Studies that allowed enrollment of participants who had previous trauma to the upper extremity, polyneuropathy, pregnancy, or diabetes will also excluded. As long as clear inclusion and exclusion criteria for healthy, asymptomatic participants are provided, the recruitment or sampling methods used by a study to enroll participants will not limit inclusion of data into the final analysis. However, studies that established a control group using the contralateral, asymptomatic hands from patients seeking care for symptoms in the opposite hand will be excluded.

#### Variable of interest

The primary variable of interest in this systematic review and meta-analysis is sonographic measurement of median nerve CSA in the distal upper extremity (i.e., elbow to hand). Surface anatomy or sono-anatomy landmarks will be used to group the CSA measurements into four primary anatomical regions:

1. Proximal forearm: > 6 cm from the distal wrist crease
2. Distal forearm/wrist: over the pronator quadratis or at the distal wrist crease
3. Proximal carpal tunnel: level of the pisiform bone
4. Distal carpal tunnel: hook of the hamate

To be included, a study must provide a clear description of the sonographic protocol for image acquisition and analysis. Image acquisition must have been completed using a linear transducer with a frequency > 10 MHz, with the participant’s arm supinated and resting in a relaxed position. Analysis of CSA on resulting images must have been completed by tracing along the inner hyperechoic border of the nerve. Data from studies measuring around the outer nerve border, fitting an ellipse to the nerve, or calculating CSA using the nerve height and width in an ellipse formula will not be included. Additional sonographic measures (e.g., elastography, nerve excursion/gliding, Doppler blood flow) will not be analyzed in this study. Secondary variables of interest for this review include sex, age, race/ethnicity, and handedness, each of which will be used to further stratify reference values of median nerve CSA in the final report as able.

### Study identification

A clinical and research librarian (LK) created and documented search strategies in the following bibliographic databases: Ovid MEDLINE, Embase, Cochrane Library, CINAHL, and SPORTDiscus on March 20, 2017. A combination of subject headings (when available) and keywords were used for the concepts of *peripheral nerves* and *reference values*, or *median nerve*, which were each combined with *ultrasonography*. Given continuous advancements in the quality of sonographic imaging acquisition of small musculoskeletal structures and techniques for image analysis, a publication date search limit was used to restrict results from 2000 to present. No language or other limits were applied. Full search strategies for each database are appended as Additional file 1 to this protocol manuscript.

Multiple efforts have been, and will be, used to minimize risk of bias within the study identification process. First, to ensure inclusion of gray literature, a search was conducted in ClinicalTrials.gov, and the investigators of any completed studies that appear to meet inclusion criteria will be contacted to obtain relevant data on healthy participants that is not identified in a published manuscript. Second, multiple hand searches will be conducted to ensure all published and non-published relevant data sources have been identified. This includes a hand search through journals and conference abstracts in the fields of sonography and nerve injury/rehabilitation, as well as a hand search through cited references of all studies identified for inclusion and relevant review articles screened through the process but not included. Finally, once the data is ready for synthesis, a rerun of the full search and study selection process will be conducted to identify and include recently published and non-published studies that meet the inclusion criteria of the review.

A review team consisting of four members with expertise in rehabilitation was formed for the study selection process. The process of decision-making for inclusion based on the eligibility criteria was piloted on a small sample of articles to validate the criteria and refine the process for study selection. Consensus meetings were conducted during the screening process and continue to occur among the review team during the full-text eligibility review to resolve ongoing discrepancies. The study selection process consists of three steps:

**Step 1: Screening of titles and abstracts**

Search results were screened at the level of title and abstract by at least two independent reviewers. Liberal general inclusion criteria were used at this initial screening level such that any articles using sonography to measure the peripheral nerves of the upper extremity were selected. Articles were not required to specifically focus on the median nerve, nor was it required that the abstract mention CSA as a study measure. Agreement between the reviewers was not required at this screening step, and any abstract selected by at least one reviewer was moved forward for eligibility review.

**Step 2: Screening of full texts for eligibility**

At this step—currently in progress—a review of the full text for each selected abstract is being completed by at least two independent reviewers to determine if the article meets all eligibility criteria. Agreement of both reviewers is required to move the study to inclusion, with a third reviewer serving as a tie-breaker. Consensus meetings among the team are being held when any reviewer is unable to make a decision regarding inclusion for an article. At this step, results from the gray literature (e.g., conference abstracts, study protocols) are being compared to other published literature to identify possible full publication of study findings. Duplicate data results are being removed, and the investigators of any unique items are being contacted in an attempt to obtain relevant data that has not been published for inclusion in the final quantitative analysis.

**Step 3: Review of eligible studies for final inclusion in quantitative analysis**

All articles deemed eligible will be reviewed one final time to ensure the participant inclusion criteria and sonographic imaging acquisition and analysis protocols meet minimum accepted quality standards. Finally, the data presented in the article will be evaluated to ensure there is sufficient information for inclusion in a quantitative analysis (i.e., averages and measures of variance). For studies meeting all inclusion criteria with exception of appropriately reported data, authors will be contacted in an attempt to obtain the required data for inclusion in the meta-analysis.

### Data management

**Fig. 1:**
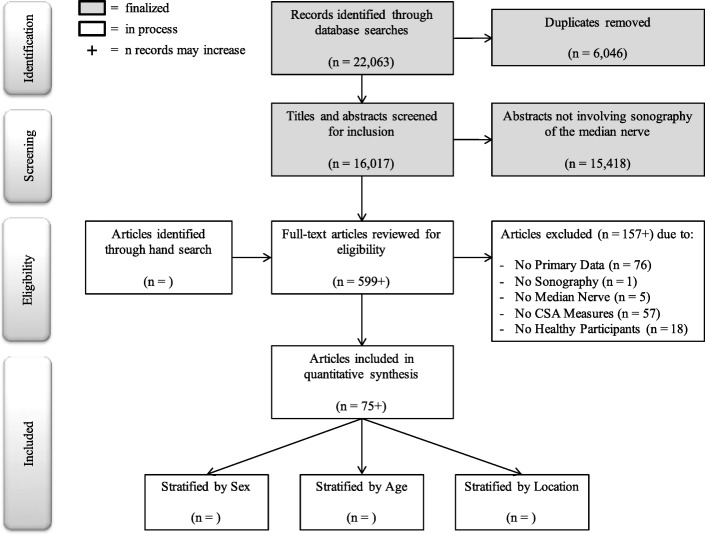
PRISMA flow diagram indicating current status and anticipated results

At the outset of this review process, all search results were compiled in EndNote and duplicates were identified. This software is being used to manage references through each step of the screening process, and archival back-up files are created with those references included at each successive stage of the review process. Each of the individual, blinded review steps are being completed using the online systematic review production software, Covidence. The PRISMA flow diagram will be used to document the number of studies moving through each step of the selection process and to identify primary reasons for study exclusion within the final step. Figure 1 provides a summary of the current status of the screening process and identifies plans for final inclusion.

### Quality assessment

For the purpose of this systematic review and meta-analysis, all included articles will be assessed for internal validity using a modified version of the National Institute of Health Study Quality Assessment Tool for Observational Cohort and Cross-Sectional Studies (https://www.nhlbi.nih.gov/health-topics/study-quality-assessment-tools). Relevant questions from the assessment tool were identified and either included verbatim or adapted to best satisfy the purpose of this review (Table 1). The general guidelines for determining the overall quality rating of these studies will be followed to rate each article’s quality. This tool will be used to identify studies for exclusion that do not meet a minimum quality reporting threshold to ensure that the final statistical analysis conducted in this review will be valid. The ratings for all included studies used in the quantitative analysis will be reported in the final publication of this review.

**Table 1:** Quality assessment tool adapted from National Institute of Health (NIH) Quality Assessment Tool for Observational Cohort and Cross-Sectional Studies

Criteria	Yes	No	Other (CD, NR, NA)	NIH Study Quality Assessment Tool Question
1. Was the research question or objective in this paper clearly stated?				1
2. Was the study population clearly specified and defined?				2
3. Were all the subjects selected or recruited from the same or similar populations (including the same time period)? Were inclusion and exclusion criteria for being in the study prespecified, rigorous, and applied uniformly to all participants?				4*
4. Was the protocol for image acquisition clearly defined (including the type of machine and appropriate transducer range > 10 MHz)?				11*
5. Was the protocol for analyzing the outcome measure clearly defined?				11*
6. Was the outcome measure collected and analyzed by a qualified person?				11*
7. Were the outcome assessors blinded to the exposure (or health) status of participants?				12
8. Were key potential confounding variables measured and adjusted statistically for their impact on the relationship between exposure(s) and outcome(s) (e.g., gender, hand-dominance)				14
Quality rating (good, fair, or poor)				
Rater #1 initials:				
Rater #2 initials:				
Additional comments (If poor, please state why):				

*CD* cannot determine, *NA* not applicable, *NR* not reported

*Adapted NIH Question

### Data synthesis

Data will be extracted from all included articles by trained reviewers and will be managed using the Research Electronic Data Capture (REDCap) system [26]. Data abstracted from the studies will include demographic information (e.g., age, sex, race/ethnicity, handedness) of the healthy, asymptomatic participants; a description of the participant inclusion and exclusion criteria;’ study sample size; study and author location (e.g., country or nation); anatomic location(s) of median nerve measurement; and the mean and variance measures of median nerve CSA.

For data deemed appropriate for quantitative synthesis, a meta-analysis will be performed. Weighted averages for the median nerve CSA will be calculated for all relevant studies, and results will be stratified by anatomic location. The meta-analysis will be performed using a random-effects model, specifying each study-specific median nerve CSA parameter as an effect size; an overall summary and study-specific effect sizes and 95% confidence intervals will be reported in forest plots for each median nerve parameter. Within anatomic location, the *I*^2^ test statistic will be used to evaluate heterogeneity of CSA means across studies. Possible small study effects will be further graphically examined by funnel plots. Studies that provide individual-level data or studies conducted on an exclusive, homogeneous population (e.g., all females) will be evaluated in separate statistical models such that results may be stratified by demographic characteristics, including age, sex, or race/ethnicity.

Because it is anticipated that the majority of studies will report mixed data from heterogeneous samples and it will not be feasible to obtain individual participant data from the large number of studies expected to be included in this review, additional statistical modeling will be conducted. Specifically, meta-regression will be used to evaluate and compare median nerve CSA parameters among different anatomic locations and among available demographic groups within the aggregate, pooled data from mixed population studies. These meta-regression results will provide estimates of the potential effects of various demographic factors on the pooled reference values. Knowledge of these effects will assist in interpretation and use of the pooled values relative to specific clinical or research needs. All statistical analyses will be carried out with STATA 15 software (StataCorp. 2017. Stata Statistical Software: Release 15. College Station, TX: StataCorp LLC).

An additional qualitative synthesis will be conducted for any included articles that are not appropriate for the full quantitative synthesis. Potential reasons for an inability of data to be used in the quantitative synthesis may include studies focused on a unique subgroup of healthy, asymptomatic participants (e.g., wheelchair users); unclear description of the anatomical location of median nerve CSA measurement; or other characteristics that render a study distinct from other studies included in the review.

### Patient and public involvement

No patients or members of the public were involved in the development of this systematic review and meta-analysis protocol. The review findings will be disseminated widely through publication in a peer-reviewed journal and conference presentations. The final findings will be shared with relevant stakeholder groups that have published practice guidelines and represent professionals who commonly utilize sonographic imaging for the evaluation of the median nerve (e.g., American College of Occupational and Environmental Medicine, American Association of Neuromuscular and Electrodiagnostic Medicine).

## Discussion

Current estimates of normal median nerve CSA stem largely from small studies that report these data from a local pool of healthy or control participants, and few studies have been solely designed to estimate normative values. This systematic review and meta-analysis uses a rigorous methodology to establish a comprehensive set of reference values of median nerve CSA across all healthy, asymptomatic populations. The review will examine published and gray literature to include all possible studies that have measured median nerve CSA in healthy, asymptomatic participants. Using this large pool of data, this study will be able to synthesize the data to establish normative median nerve CSA values that can be used to guide and standardized future research and clinical practice.

This rigorous protocol was developed to include all possible available data and provide the most accurate estimate of median nerve CSA values across multiple populations. By rerunning the search to also include all current studies and completing additional hand searches of review article references and selected journals, this meta-analysis seeks to be inclusive of all published and non-published literature. This analysis will also include an additional step in thoroughness by contacting investigators of studies for which data have not been published and publications with insufficient data reported.

Substantial missing data or unclear information (e.g., demographic characteristics of healthy participants) is the primary risk of bias or limitation in reporting for this review. Missing data may limit the analysis of CSA measures for heterogeneous groups of asymptomatic, healthy populations. However, this risk is considered minimal, as the current process has already resulted in an adequate number of studies that meet the final inclusion criteria, despite only completing a small portion of the full-text reviews. A more likely potential limitation of this review will be an inability to provide strong evidence across the various anticipated stratifications. It is likely that data will be able to be presented by sex and anatomic location; however, data will likely need to be stratified across relatively large age ranges and the ability to stratify results by race/ethnicity and handedness will likely be more limited.

While the power to provide rigorous data for some sub-stratifications may be limited, providing generalized reference values and stratified values for key descriptive variables will prove to be a substantial asset to the scientific and clinical communities. Studies that utilize these reference values can more easily be compared, and true variability in diagnostic accuracy, diagnostic thresholds, and other diagnostic parameters using median nerve CSA can be clarified. Moreover, these normative values will provide a foundation upon which studies can further investigate preclinical stages of median nerve pathology, providing a means to understand minor nuances in changes to the CSA as related to the development of symptoms. Finally, these values can serve as a resource for evaluating meaningful clinical differences and other changes to tissue morphology that may be useful in evaluating outcomes of clinical interventions for median nerve pathology.

## Additional file

Additional file 1: Search strategies. (PDF 84 kb)

## Abbreviations

CSA: Cross-sectional area
PRISMA-P: Preferred Reporting Items for Systematic Reviews and Meta-Analyses Protocols
PROSPERO: International Prospective Register of Systematic Review;
REDCap: Research Electronic Data Capture
