# Supplementary material for: Sonographic reference values of median nerve cross-sectional area: a protocol for a systematic review and meta-analysis
Source: Syst Rev. 2019 Jan 3;8:2. doi: 10.1186/s13643-018-0929-9 (PMC6317213; doi:10.1186/s13643-018-0929-9)
Supplement: Supplementary file 1 — Search strategies. (PDF 84 kb) [file 13643_2018_929_MOESM1_ESM.pdf]

SUPPLEMENT: SEARCH STRATEGIES

Database Names: Ovid Medline® Epub Ahead of Print, In-Process & Other Non-Indexed Citations, Ovid Medline® Daily, Ovid Medline®
Database Vendor: Wolters Kluwer
Database Coverage: 1946 – Present
Date Last Searched: March 20, 2017

((exp Peripheral Nervous System/ OR peripheral nerve.af OR peripheral nerves.af) AND (exp Reference Values/ OR (reference ADJ3 (value OR values)).af)

OR

exp Brachial Plexus/ OR exp Median Neuropathy/ OR exp Neural Conduction/ OR (median ADJ3 (nerve OR nerves)).af OR carpal tunnel.af)

AND

((elastic* ADJ3 (image OR images OR imaging OR imagings)).af OR exp Ultrasonography/ OR exp Electrodiagnosis/ OR exp Elasticity/ OR ultrason*.af OR echograph*.af OR ultrasound.af OR ultrasounds.af OR sonograph*.af OR electrodiagnosis.af OR electrodiagnoses.af OR elastogram*.af OR elestograph*.af)

Limit to Publication Year: 2000-current

Database Name: Embase & Embase Classic

Database Vendor: Elsevier
Database Coverage: 1947 – Present
Date Last Searched: March 20, 2017

('peripheral nervous system'/exp OR (peripheral AND (nerve OR nerves)) AND ('reference value'/exp OR reference NEAR/3 (value OR values))

OR

'brachial plexus'/exp OR 'carpal tunnel syndrome'/exp OR 'nerve conduction'/exp OR median NEAR/3 (nerve OR nerves) OR (carpal AND tunnel))

AND

('echography'/exp OR 'electrodiagnosis'/exp OR 'elasticity'/exp OR ultrason* OR echotomograph* OR echograph* OR ultrasound OR ultrasounds OR sonograph* OR electrodiagnosis OR electrodianoses OR elastogram* OR elastograph* OR (elastic* NEAR/5 (image OR images OR imaging OR imaging)))

AND

[2000-2017]/py

Database Name: Cochrane Library
Database Vendor: Wiley
Issues Searched: Cochrane Reviews (Issue 3 of 12, March 2017)
Other Reviews (Issue 2 of 4, April 2015)
Trials (Issue 2 of 12, February 2017)
Methods Studies (Issue 3 of 4, July 2012)
Technology Assessments (Issue 1 of 4, January 2016)
Economic Evaluations (Issue 2 of 4, April 2015)
Date Last Searched: March 20, 2017

#1	MeSH descriptor: [Peripheral Nervous System] explode all trees
#2	peripheral nerve
#3	peripheral nerves
#4	#1 OR #2 OR #3
#5	MeSH descriptor: [Reference Values] explode all trees
#6	reference
#7	value
#8	values
#9	#7 OR #8
#10	#6 AND #9
#11	#5 OR #10
#12	#4 AND #11
#13	MeSH descriptor: [Brachial Plexus] explode all trees
#14	MeSH descriptor: [Median Neuropathy] explode all trees
#15	MeSH descriptor: [Neural Conduction] explode all trees
#16	median
#17	nerve
#18	nerves
#19	#17 OR #18
#20	#16 AND #19
#21	carpal tunnel
#22	#13 OR #14 OR #15 OR #20 OR #21
#23	elastic
#24	image
#25	images
#26	imaging
#27	imagings
#28	#24 OR #25 OR #26 OR #27
#29	#23 AND #28
#30	MeSH descriptor: [Ultrasonography] explode all trees
#31	MeSH descriptor: [Electrodiagnosis] explode all trees
#34	MeSH descriptor: [Elasticity] explode all trees
#35	ultrason*
#36	echotomograph*

#37 echograph*
#38 ultrasound
#39 ultrasounds
#40 sonograph*
#41 electrodiagnosis
#42 elastogram*
#43 elastograph*
#44 #29 OR #30 OR #31 OR #32 OR #33 OR #34 OR #35 OR #36 OR #37 OR #38 OR #39 OR #40 OR #41 OR #42 OR #43
#45 #12 OR #22
#46 #44 AND #45

Removed citations outside of 2000-present date range with EndNote X8 software

---

Database Name: CINAHL Complete
Database Vendor: EBSCO
Database Coverage: 1937 – Present
Date Last Searched: March 20, 2017

(((MH "Peripheral Nervous System+" OR peripheral nerve) AND (MH "Reference Values" OR reference values))

OR

(MH "Brachial Plexus+" OR MH "Carpal Tunnel Syndrome" OR MH "Neural Conduction" OR (median N3 nerve) OR carpal tunnel))

AND

((elastic* AND imag*) OR MH "Ultrasonography+" OR MH "Electrodiagnosis+" OR MH "Elasticity+" OR ultrason* OR echotomograph* OR echograph* OR ultrasound OR sonograph* OR electrodiagnosis OR electrodiagnoses OR elastogram* OR elastograph*)

Limiters - Published Date: 20000101-20171231

Database Name: SPORTDiscus
Database Vendor: EBSCO
Database Coverage: 1892 – Present
Date Last Searched: March 20, 2017

(((DE "PERIPHERAL nervous system" OR DE "SPINAL nerves" OR peripheral nerve) AND reference values)

OR

DE "BRACHIAL plexus" OR DE "MEDIAN nerve" OR DE "RADIAL nerve" OR DE "ULNAR nerve" OR DE "CARPAL tunnel syndrome" OR DE "NEURAL conduction" OR median nerve OR carpal tunnel)

AND

((elastic* AND imag*) OR DE "ULTRASONIC imaging" OR DE "DIAGNOSTIC ultrasonic imaging" OR DE "ELASTICITY (Physiology)" OR ultrason* OR echotomograph* OR echograph* OR ultrasound OR sonograph* OR electrodiagnosis OR elastogram* OR elastograph*)

Limiters - Published Date: 20000101-20171231
